# Diel patterns of microphytobenthic primary production in intertidal sediments: the role of photoperiod on the vertical migration circadian rhythm

**DOI:** 10.1038/s41598-019-49971-8

**Published:** 2019-09-16

**Authors:** S. Haro, J. Bohórquez, M. Lara, E. Garcia-Robledo, C. J. González, J. M. Crespo, S. Papaspyrou, A. Corzo

**Affiliations:** 1 Department of Biology, University of Cádiz, Puerto Real, 11510 Spain; 2 Division of Naval Support and Oceanography, Marine Hydrographic Institute, Spanish Navy, Cadiz, Spain; 3 Instituto Universitario de Investigación Marina (INMAR), Universidad de Cádiz, Campus de Excelencia Internacional del Mar (CEIMAR). Campus Universitario de Puerto Real, Puerto Real (Cádiz), 11510 Spain

**Keywords:** Microbial ecology, Marine biology

## Abstract

Diel primary production patterns of intertidal microphytobenthos (MPB) have been attributed to short-term physiological changes in the photosynthetic apparatus or to diel changes in the photoautotrophic biomass in the sediment photic layer due to vertical migration. Diel changes in primary production and vertical migration are entrained by external factors like photoperiod and tides. However, the role of photoperiod and tides has not been experimentally separated to date. Here, we performed laboratory experiments with sediment cores kept in immersion, in the absence of tides, with photoperiod or under continuous light. Measurements of net production, made with O_2_ microsensors, and of spectral reflectance at the sediment surface showed that, in intertidal sediments, the photoperiod signal was the major driver of the diel patterns of net primary production and sediment oxygen availability through the vertical migration of the MPB photoautotrophic biomass. Vertical migration was controlled by an endogenous circadian rhythm entrained by photoperiod in the absence of tides. The pattern progressively disappeared after 3 days in continuous light but was immediately reset by photoperiod. Even though a potential contribution of a subjective *in situ* tidal signal cannot be completely discarded, Fourier and cross spectral analysis of temporal patterns indicated that the photosynthetic circadian rhythm was mainly characterized by light/dark migratory cycles.

## Introduction

Intertidal sediments are a complex environment where strong physicochemical changes occur at different spatiotemporal scales, i.e. diel photoperiod and tidal cycles, fortnight tidal cycles (spring-neap tides), and seasonal changes^1^. Microorganisms inhabiting the sediment phase their biological activities to this environmental variability to foster their survival and growth^2–4^. One such example is microphytobenthos (MPB), i.e. the community of microbial primary producers inhabiting intertidal sediments. These organisms, mainly diatoms and cyanobacteria, have important ecological and biogeochemical roles^5–8^ and contribute significantly to the total primary production (PP) in shallow coastal environments^9,10^. Microphytobenthic PP is expected to change during the daylight as a consequence of the diel changes in solar elevation and consequently irradiance from dawn to dusk^11,12^. In addition, intertidal MPB PP is largely controlled by tides, with maximum rates being frequently observed during low tide^13–15^. However, the specific mechanism and how photoperiod and tide interact to determine the rate of *in situ* PP at different time scales is largely unknown^16,17^.

Diel changes of photosynthesis rate in many primary producers, from cyanobacteria to higher plants are endogenously controlled by circadian rhythms, endogenous biological clocks that time metabolic, physiological and behaviour events to the diel cycle^18–22^. A biological rhythm is controlled by a circadian clock when (1) it has a periodicity of about 24 h; (2) persists for several days in the absence of the stimulus that triggers it (free-running rhythm); (3) the period of the free-running rhythm is not exactly 24 h; and (4) it can be reset^23^. The existence of a circadian rhythm in the photosynthetic activity increases growth, survival and competitive fitness in primary producers^24–26^. Photosynthetic circadian rhythms are controlled through the regulation of different components of the photosynthetic apparatus (i.e. stomatal opening, intermediates of Calvin cycle, photosynthetic pigments levels, activity of photosystem II, chloroplast movements and gene transcription of proteins that regulates specific photosynthetic processes) in different species and taxonomic groups^19,21,27–29^. Unfortunately, our knowledge of the importance of all these potential physiological-genomic regulation mechanisms of the diel photosynthetic rhythm in MPB species is very scarce. Diel changes in MPB photosynthetic parameters, frequently observed in photosynthetic efficiency (α), maximum quantum yield (F_v_/F_m_) and maximum photosynthesis rate (P_max_) with the Pulse Amplitude Modulated (PAM) fluorescence technique, have been attributed to several mechanisms acting, at a biochemical or physiological level, on processes associated to the light and dark reactions. These include changes in light-harvesting complexes, non-photochemical quenching, efficiency of energy transfer from the light-harvesting antennae to the reaction centers, the number of functional PSII reaction centers, state of the xanthophyll cycle, and activity of some Calvin cycle enzymes^30–33^.

Diel vertical migration of benthic microalgae has been known for a long time^34,35^, however, its purpose is still under debate. In addition to being a behavioural photoprotection mechanism^15,36–38^, other suggested causes of vertical migration include avoidance of resuspension, reduced grazing pressure, higher nutrient availability, and environmental stability for cellular division in deeper sediment layers^2,15^. Independently of its cause or adaptive purpose, the upward and downward vertical displacement of the photoautotrophic biomass within the sediment is probably a major determinant of the diel rate of primary production - in addition to physiological photoadaptation and photoacclimation mechanisms in action as a response to the changing irradiance during the day. However, the relative importance of vertical migration and photophysiology seems to be different, depending on species, growth form, and environmental conditions^31–33,38–41^.The existence of diel vertical migration of photoautotrophic biomass in systems without tidal signals, freshwater sediment^34,42^, and their relatively recent discovery in subtidal marine sediment^12,43^ suggest that light and tidal cues can operate independently and that, in intertidal sediments, the photoperiod might be the major environmental driver of vertical migrations, with tides being an additional secondary environmental cue. The way the coupling between photoperiod and tidal signals occurs is not known. Due to the co-occurrence of both signals in intertidal areas, *in situ* studies cannot unambiguously distinguish between their respective contributions to the observed MPB primary production and vertical migration patterns. In addition, most of the laboratory experiments have not tried to distinguish clearly between both signals.

In this study, we show that diel oscillations of net primary production in intertidal sediments, measured by O_2_ microsensors, can occur in the absence of tides due to the vertical migration of photoautotrophic biomass (estimated by spectral reflectance). This migration is under the control of a circadian rhythm entrained by the photoperiod signal, even though the contribution of other environmental clues, like the subjective tidal cycle cannot be potentially discarded. This oscillatory diel pattern in primary production has practical implication for the precise measurement of daily and seasonal rates and it has also deep ecological and biogeochemical implications, regarding the coupling between the photoautotrophic and heterotrophic communities, the sediment net metabolism and the rate and pathway of organic matter mineralization.

## Results

### Diel patterns of net production and respiration

**Figure 1:**
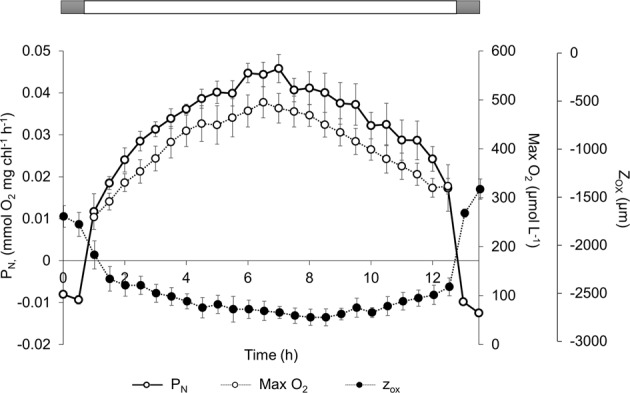
Temporal evolution of net primary production in the photic zone (P_N_; continuous line), maximum oxygen concentration (Max O_2_; discontinuous line with open symbols) and maximum oxygen penetration depth (z_ox_; discontinuous line with filled symbols) in submerged intertidal sediment during the light phase of the photoperiod. The horizontal white bar indicates the light phase (12 h) at a constant irradiance of 200 µmol photon m^−2^ s^−1^. Values are means (n = 6) ± standard error.

**Figure 2:**
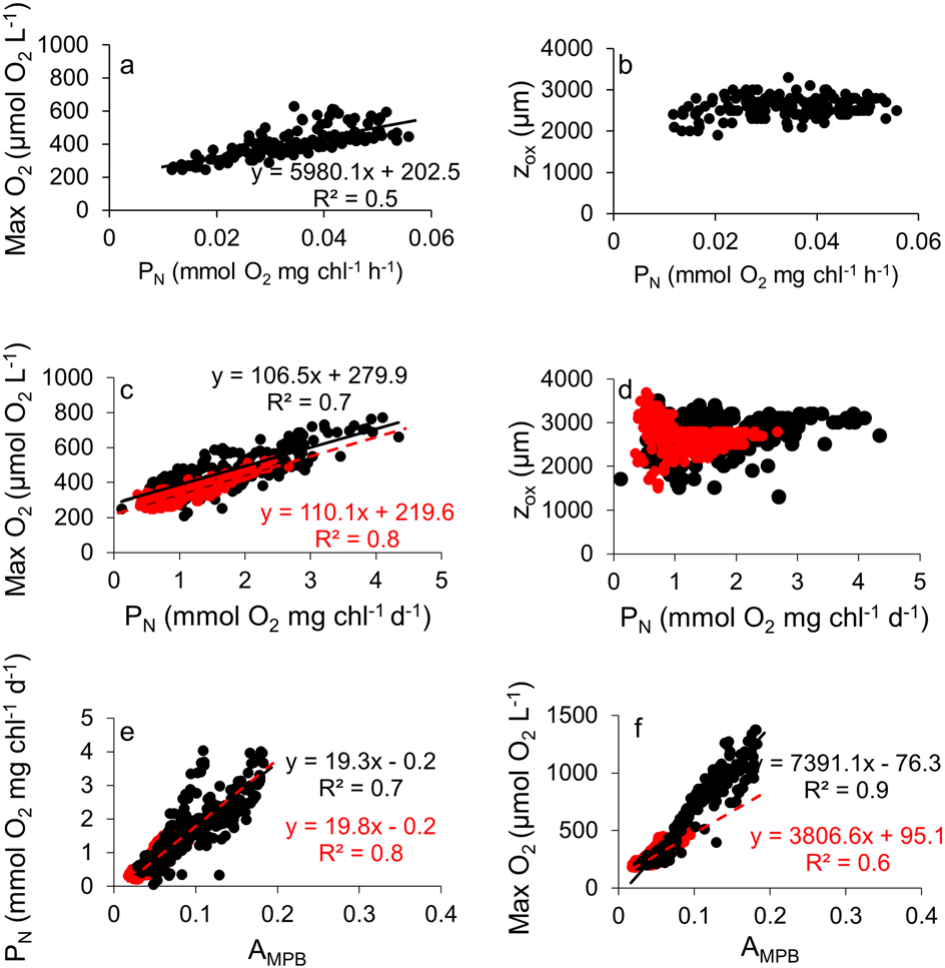
(**a,b**) Relationship between the maximum O_2_ concentration (Max O_2_) and O_2_ penetration depth (z_ox_) within the sediment with net production in the photic layer (P_N_) during the light and dark periods of a single day (data from ExI). (**c,d**) Max O_2_ and z_ox_ as a function of P_N_ in photoperiod and in continuous light (data from ExII). (**e**,**f**) P_N_ and Max O_2_ as a function of the light absorbed by microphytobenthos (A_MPB_), used as a proxy of MPB biomass in the upper sediment layer (data from ExIII). Black and red symbols indicate photoperiod and continuous light, respectively.

Sediment profiles of O_2_ in the absence of any tidal stimulus changed considerably along the day under a 12 h light: 12 h dark (12 L:12D) photoperiod (Supplementary Material, Fig. S2). During the dark period, O_2_ was consumed within the sediment at a constant rate (0.01 ± 0.0007 mmol O_2_ mg Chl^−1^ h^−1^, F_1,5_ = 1.95; p = 0.15; one-way ANOVA) with the maximum O_2_ penetration depth (z_ox_) being only 1.5 mm (Fig. 1; Supplementary Material, Fig. S2). During the light period, microphytobenthic net production in photic layer (P_N_) increased initially (0.02 mmol O_2_ mg Chl^−1^ h^−1^) up to a maximum between 5–9 hours (0.06 mmol O_2_ mg Chl^−1^ h^−1^) and then decreased. The same general pattern was observed in all 6 independent replicates (Fig. 1). Maximum oxygen concentration (Max O_2_) was 400–500 µmol O_2_ L^−1^ and z_ox_ 2.5 mm (Fig. 1; Supplementary Material, Fig. S2). Max O_2_ and z_ox_ followed the pattern of net primary production in the photic zone (Fig. 1). Max O_2_ was linearly correlated with the P_N_ (r = 0.74; p < 0.05; n = 140), but z_ox_ was less affected by increases in P_N_ (r = 0.25; p < 0.05; n = 140) (Fig. 2a,b).

### Importance of the photoperiod signal to the diel patterns of net production and respiration

**Figure 3:**
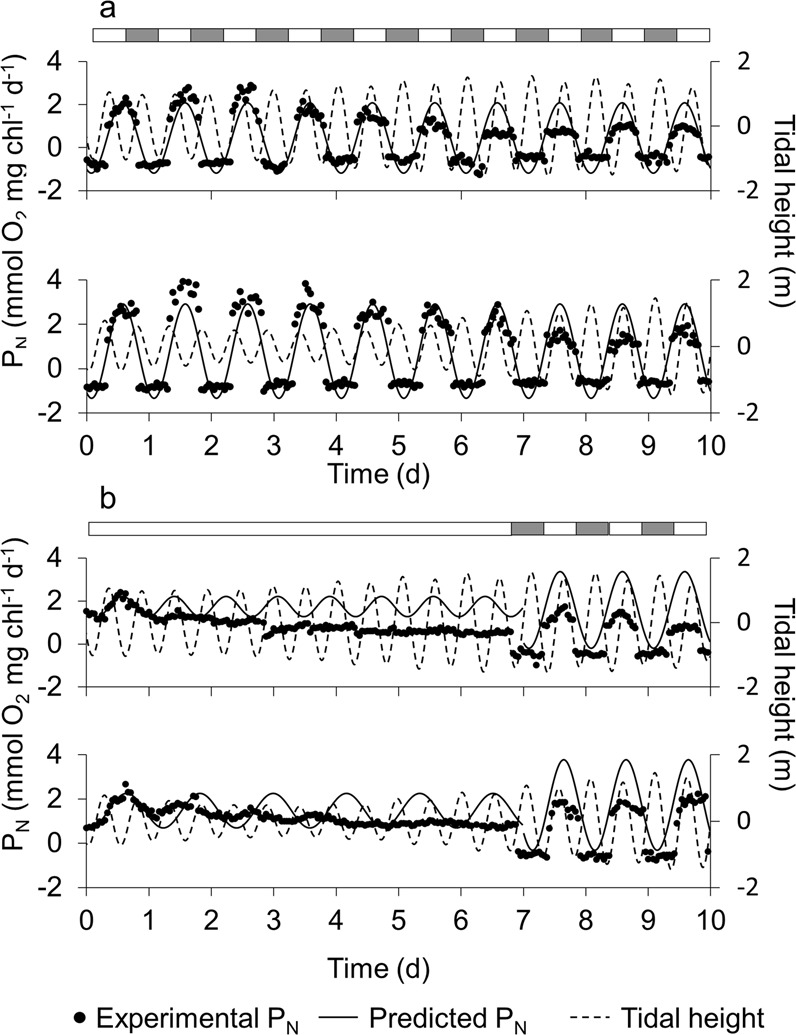
Examples of (**a**) daily net production (P_N_) patterns of MPB during 10 days under 12 h light: 12 h dark photoperiod and (**b**) in continuous constant light for 7 days, followed by a 12 h light: 12 h dark photoperiod rhythm for the last 3 days. Irradiance during the light phase was always 200 µmol photon m^−2^ s^−1^. Black symbols are experimental data of P_N_, the continuous line is the predicted data of microphytobenthic net production (Eq. 6) and the discontinuous line is the *in situ* tidal height at the sampling site for days of the experiment. Given the evident dampening of experimental data under continuous light, we only used the first 24 h under continuous light to obtain the sine wave function, while we used the entire time series (10 days) under the photoperiod. Grey and white bars indicate dark and light phases, respectively.

**Figure 4:**
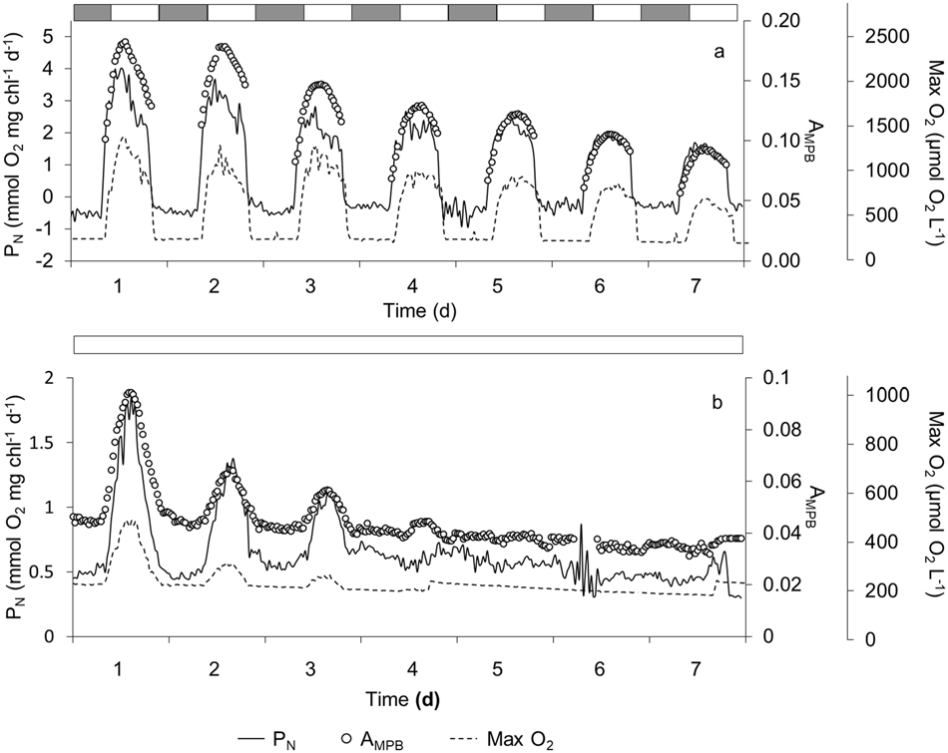
Examples of net primary production by microphytobenthos (continuous line), light absorbed by microphytobenthos (A_MPB)_ determined by reflectance spectra (open symbol) and maximum oxygen concentration (discontinuous line) under (**a**) a 12 h light: 12 h dark photoperiod and (**b**) continuous light for 7 days. Irradiance during the light phase was 200 µmol photon m^−2^ s^−1^. Grey and white bars indicate dark and light phases, respectively. This experiment was repeated twice with similar results.

The typical diel pattern in P_N_ during the light phase of the photoperiod was kept until the end of several independent experiments conducted for 7–10 days (Figs 3a and 4a). However, this pattern disappeared under continuous light after 2–4 days (Figs 3b and 4b). In addition, maximum levels of P_N_ tended to decrease along the experiments both in the presence of photoperiod and under continuous light but with a different trend. P_N_ decayed linearly under a photoperiod and logarithmical under the continuous light treatment (r > 0.97, p < 0.05, n = 7 in both cases) (Supplementary Material, Fig. S3). The diel P_N_ cycle quickly recovered after 12 hours in the dark and the photoperiod re-established. P_N_ reached levels similar to those at the beginning of the experiment and significantly higher than those observed at the end of the continuous light treatment (days 5–7) (Fig. 3b). Contrary to P_**N**_, respiration rate in darkness (R_d_) remained rather constant over the night period under a 12 L:12D photoperiod (average R_d_ = −0.44 ± 0.19 mmol O_2_ mg chl^−1^ d^−1^; n = 200) (Figs 3a and 4a). The difference in the response of P_N_ and R_d_ to light conditions along the diel photoperiod cycle induced important changes in the oxygen concentration within the sediment. Max O_2_ correlated linearly with P_N_ under both the 12 L:12D photoperiod (r = 0.81; p < 0.05; n = 315) and under continuous light (r = 0.88; p < 0.05; n = 519) in a similar way (Fig. 2c), whereas z_ox_ did not (Fig. 2d).

**Figure 5:**
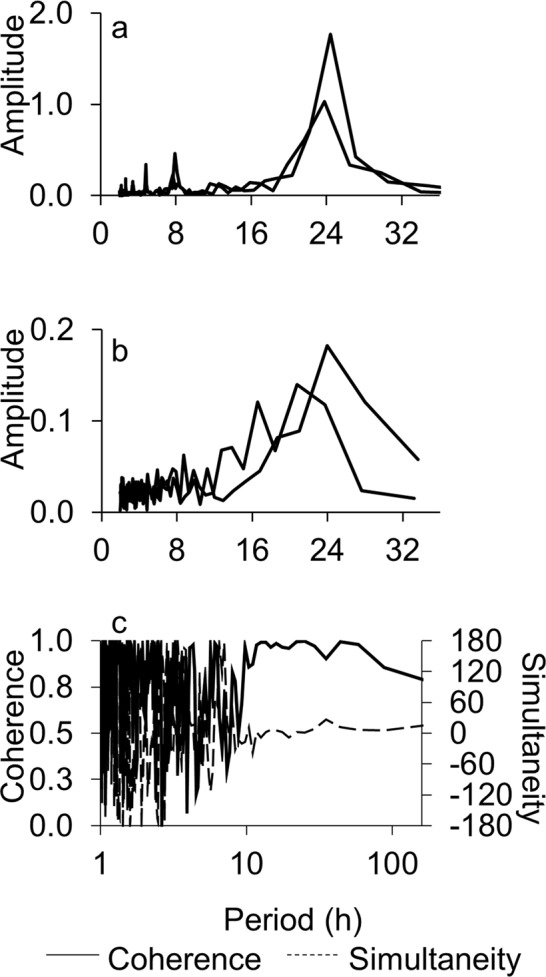
Relationship between amplitude of the signal and different periods determined by Fourier spectral analysis from the net primary production (P_N_) time series under (**a**) 12 h light: 12 h dark cycles and (**b**) continuous light. (**c)** Changes in coherence (continuous line) and simultaneity (broken line) with time along the experiment were determined by cross spectral analysis between P_N_ and and A_MPB_ time series in continuous light. Period or frequency are represented on a logarithmic scale. Coherence values close to 1 indicate a high spectral correlation, i.e. a strong temporal covariation between both data series. Simultaneity values close to 0 indicate that both variables are nearly in phase for a given temporal frequency.

The predicted sine equation that best fitted to the P_N_ experimental time series during the first 24 h allowed the comparison of the P_N_ evolution with its “reference” level at the beginning of the experiments (Fig. 3). Under constant light, the amplitude of the P_N_ oscillation were about only 33% of those under a 12 L:12D photoperiod. The period in continuous light was 19.9 and 28.2 h in four different experiments, while under the experimentally imposed photoperiod the period was as expected 24 h (Supplementary Material, Table S1). To analyse the presence of possible masked tidal signals in the MPB community as a possible endogenous rhythm, due to semidiurnal or fortnight spring-neap tidal variation, we used two approaches. In the first, we compared the peak in P_N_ to the *in situ* tidal stage on the days of the experiment (broken line Fig. 3). Maximum P_N_ tended to decrease as the difference between the time at which the maximum P_N_ was observed in the laboratory and the time of maximum low tide *in situ* increased; however, this trend was not statistically significant (Supplementary Material, Fig. S4). In the second approach, we applied Fourier spectral analysis (FSA) to detect any relevant time frequencies in the P_N_ time series under photoperiod and in continuous light. The FSA showed three dominant frequencies at 8 (only observed under photoperiod), 12 (semidiurnal) and 24 (diurnal) hours (Fig. 5a,b). However, only the effect of diurnal frequency was significant. Further mathematical analysis of the potential contribution of the 8 and 12 h frequencies to the P_N_ temporal patterns in the laboratory revealed that they cannot be distinguished from a mathematical effect (supplementary material).

### Relationship between the daily changes in net primary production and the light absorbed by microphytobenthos

Light absorbed by the MPB (A_MPB_**)** used as a proxy of MPB abundance at the sediment surface, increased during the first hours of the light phase of photoperiod, reaching its maximum simultaneously with the maximum P_N_ and Max O_2_ and decreasing during the last hours of the light phase (Fig. 1). This pattern was maintained throughout the 7 days of photoperiod. However, in parallel with P_N_ and Max O_2_, the maximum A_MPB_ decreased progressively from 0.2 to 0.1 during the experiment. Max O_2_ (r = 0.95; p < 0.001; n = 315) and P_N_ (r = 0.84; p < 0.001; n = 315) were linearly correlated with A_MPB_ (Fig. 2e,f). The daily oscillation in A_MPB_ was also maintained during the first days under continuous light (Fig. 4b). A_MPB_ decreased steadily for the first 3 days and remained constant after 3–4 days. Both P_N_ (r = 0.88; p < 0.001; n = 521) and Max O_2_ (r = 0.79; p < 0.001; n = 521) correlated with A_MPB_ under continuous light as well (Fig. 2e,f). Cross spectral analysis (CSA) demonstrated a high coherence and nearly in-phase relationships (simultaneity) between P_N_ and A_MPB_ for periods of variability longer than 12 hours (Fig. 5c).

## Discussion

Diel P_N_ patterns of intertidal MPB can be caused by physiological changes in the photosynthetic apparatus or by diel changes in the MPB biomass in the sediment photic layer due to vertical migration driven by photoperiod and tidal stage. However, the respective contributions of both signals cannot be unambiguously distinguished in *in situ* studies due to their co-existence in intertidal areas. Even though the potential contribution of the subjective *in situ* tidal signal cannot be irrefutably discarded, in this study, we show that in the experimentally induced absence of tides, diel changes in P_N_ are determined by vertical migration of MPB, which in turn responds to a circadian rhythm entrained by photoperiod.

During the light phase of the photoperiod at constant irradiance, MPB P_N_ changed, reaching its maximum rate after 6 hours of light (Fig. 1), as observed previously under a 12 L:12D photoperiod^11,43,44^. However, maximum oxygen concentrations were observed just two hours after the on-set of the light phase when it was set at only 5 hours^45^. This suggests that the time needed to reach the maximum P_N_ might be related to the duration of the light phase. One major difference between the results reported here and previous studies is that our results clearly demonstrated that these changes in P_N_ can be produced by a photoperiod signal since the experiments were done in immersion and in absence of tides. The intertidal sediment used in the experiments harboured a representative net autotrophic MPB community, with a mean P_N_:R_d_ ratio of 3.66 ± 0.66. P_N_ (2.69 ± 0.16 mmol O_2_ m^−2^ h^−1^) during the daily light period and R_d_ values (0.82 ± 0.16 mmol O_2_ m^−2^ h^−1^) reported here are similar to previous measurement in Cadiz Bay^46–48^ and elsewhere^9,11,43,49,50^.

The existence of P_N_ oscillations in constant light in the laboratory raises important biological and biogeochemical questions. Firstly, the daily oscillation in microphytobenthic P_N_ observed *in situ* under the natural changes in solar elevation, and consequently of irradiance, in addition to a direct response to the changes in irradiance during the daylights, could include an endogenous rhythm component. In this case, this endogenous component should be taken into account; otherwise, modelling of the hourly changes in P_N_ using a typical Photosynthesis-Irradiance curve^51^ might not be realistic enough, at least in muddy sediments dominated by epipelic diatoms^52^. Secondly, during our experiments, P_N_ increased 3.9–10.4 times during the light period under constant light. This variability in a single light period represents up to 20% of the P_N_ variability measured during an annual cycle in the same area^48^. In addition, the moment of the day when P_N_ measurements were done changed up to a 30% the estimated daily rates and therefore can affect considerably seasonal trends and annual budgets when diurnal short-term variability is not precisely measured^12,49,53^. Therefore, the endogenous diel oscillation in P_N_ must be taken into account in future *in situ* studies, although it is evident that this will represent an important logistic effort and require to complement experimental observations at higher time resolution with modelling^14,53,54^. Finally, the daily oscillations in P_N_ are likely to have strong implications on the sediment biogeochemical cycling of nutrients and the coupling between the photoautotrophic and heterotrophic sediment communities^55^. Most likely, the availability of organic substrate will follow a daily dynamic similar to P_N_ affecting the activity of the microbial heterotrophic community^11,56–58^. In addition, the changes in O_2_ availability during the light period, both in terms of Max O_2_ and z_ox_ (Figs 1, 2 and 4), can largely alter the availability of alternative electron donors and acceptors and consequently the relative contribution of oxic and anoxic mineralization near the sediment surface^4,59–61^ and the net exchange of solutes across the sediment-water interface^55^. Evidently, larger differences are expected between day and night.

MPB seems to detect both the photoperiod and the tidal signals and adjust its activity to the daily, fortnightly and seasonal environmental changes^17,35,52,62,63^. However, to investigate the relative contribution of each of the photoperiod and tide, and their specific characteristics, it is important to separate experimentally both signals. In our experiment, the diel P_N_ pattern was maintained under 12 L:12D photoperiod for as long as 10 days (Figs 3a and 4a). Similar oscillations have been observed in the laboratory^13,14,44^ and *in situ* studies^14,49,53^, but in presence of both the tidal and the photoperiod signals. Our results, on the other hand, demonstrate that the photoperiod signal is enough to keep the diel cycle of primary production in intertidal MPB. Similar results have been reported for subtidal MPB communities^12,43^. In our experiment, the P_N_ diel pattern was maintained under continuous light during several days (3–5 days), suggesting the existence of an endogenous control in the absence of a photoperiod signal as previously suggested^17,35,52,63^. Under constant light, a free-running P_N_ cycle with a period of around 24 h (between 19.91 and 28.17 h; n = 4) and a decrease in amplitude with time were observed as expected (Fig. 5; Supplementary Material, Table S1). After 7 days in continuous constant light, diel oscillations in P_N_ disappeared entirely. However, a single dark period of 12 h was enough to restore the pattern (Fig. 3b), demonstrating the importance of the dark phase in resetting the photoperiod cycle^44^.

Under constant light, the oscillation in P_N_ occurred initially over a higher basal level of P_N_ (1.28 ± 0.40 mmol O_2_ mg Chl^−1^ h^−1^) compared with the photoperiod treatment (0.82 ± 0.29 mmol O_2_ mg Chl^−1^ h^−1^) which included a dark period, where P_N_ was negative (Supplementary Material, Table S1). In both cases, average P_N_ decreased along the experiments (Figs 3 and 4), logarithmical under continuous light and linearly under light-dark cycles (Supplementary Material, Fig. S3), coinciding with previous results^44^. The damping of P_N_ daily oscillation over time was expected under constant light since the progressive suppression of the oscillation in the absence of the periodic cue is characteristic of a circadian rhythm. However, the decrease in P_N_ under the photoperiod treatment might also be due to a general decrease of MPB biomass and primary production under the laboratory experimental conditions. First, the lower light irradiance intensity in the laboratory with respect to *in situ* conditions likely decreased the growth rate and MPB biomass. Second, despite the frequent renewal of the tank water, nutrient limitation might also limit MPB P_N_ in the laboratory^10,15^.

One important aspect to consider is that an endogenous fortnightly behaviour synchronized with spring-neap tidal cycles *in situ* might induce changes in P_N_ during the experiments even in the absence of tides in the laboratory^13,14^. The numerical and statistical analysis of the P_N_ temporal pattern, by analysing the degree of coincidence between the maximum daily P_N_ and the *in situ* low tide and by applying FSA to the P_N_ temporal series were unable to identify any clear and statistically significant tidal signal (Figs 3 and 5, supplementary material). FSA picked three possible dominant frequencies at 8, 12 and 24 hours under photoperiod (Fig. 5a). The 24 h frequency in the photoperiod treatment is expected due to the experimentally imposed 12 L:12D photoperiod. These dominant frequencies were generally also observed in continuous light as well; however, the diurnal (24 h) frequency was more variable as it usually occurs during a free-running cycle in the absence of external signal (Fig. 5b)^19^. The semidiurnal (12 h) frequency in P_N_ - tides are semidiurnal in Cadiz Bay - could be a “memory” effect of the *in situ* tidal phase, despite the absence of any experimentally imposed tidal signal during the experiments. Comparison of the amplitude ratios between semidiurnal to diurnal contributions under either continuous light or photoperiod treatments (produced by Eqs S13 and S14, supplementary material) and our experimental observations suggests that the magnitude of the semidiurnal frequency cannot be distinguished from an effect produced purely by the mathematical properties of the P_N_ curves and their effects on the coefficients computed from the FSA^64^. A similar conclusion was obtained after analysing the 8 h frequency, which in addition only appeared clearly in the photoperiod treatment as predicted (Supplementary Material, Eq. S13). Moreover, the amplitude of the diurnal signal (24 h) was 6 times higher than the amplitude of the semidiurnal signal (12 h) in the photoperiod treatment (Fig. 5a,b). Therefore, our results suggest that the photoperiod signal is the main driver of the daily oscillations in P_N_ and that the associated circadian rhythm has an endogenous component. Unfortunately, the potential role of tidal signals (immersion-emersion and neap/spring tides) on the vertical migration circadian rhythm in our system cannot be entirely excluded with our experimental design. Further, the experimental separation of photoperiod and tides related signals is extremely complicated, for instance determining a potential “memory” of the *in situ* spring-neap tidal cycles in MPB kept in the laboratory^13,14^ is difficult because it would require experiments lasting more than 15 days, where potential changes in the MPB community and physiological adaptations to laboratory conditions would likely complicate interpretation of the results.

The MPB biomass close to the sediment surface was estimated from A_MPB_ determined from reflectance spectra^17,38,43^. The P_N_ and A_MPB_ temporal patterns clearly showed a strong positive covariation under both the photoperiod and continuous light treatments (Figs 2e,f and 4), as shown previously for subtidal MPB in constant light^43^. In addition, the periodicity and damping of the P_N_ and migration vertical patterns were similar under both conditions (Fig. 4). This strongly supports the hypothesis that the observed daily oscillation in the P_N_ rate in muddy sediments is caused by the vertical migration of MPB, whereas the observed damping of the P_N_ oscillation with time in the laboratory is most likely the consequence of a progressive decrease in the number of cells that migrate upward during the light phase^31,32,39^. CSA between P_N_ and A_MPB_ time series under continuous light - this analysis was not possible for the photoperiod time series since we lack reflectance data during the dark period - corroborated the strong covariation between both variables, showing a high coherence and nearly in-phase relationships between their temporal patterns for periods longer than 12 h (Fig. 5c). Therefore, P_N_ increases during daylight due to the progressive accumulation of cells at the sediment photic layer as a result of upward vertical migration. It reaches a maximum coinciding with the maximum accumulation of cells in the sediment photic layer and finally, during the last hours of the light phase, P_N_ begins to decrease due to the downward migration of cells below the photic layer, where they remain during the dark period. It is unclear what the purpose of maintaining a vertical migratory circadian rhythm in our experimental conditions is. In emersion, the downward migration during the light period is considered a behavioural photoprotection mechanism against increasing light dose^39^ or the result of endogenously controlled positive geotaxis^17,65^, but in our experiments sediment cores were always kept in immersion and at low light irradiance. Alternatively, cytological analysis of diatom distribution shows that the proportion of cells in mitosis increases with increasing depth^2^. Epipelic diatoms could divide at night in deeper sediment layers due a to a higher nutrient availability and higher environmental stability and migrate upward during the day to collect light energy^2,15,57^. Independently of the reason for the daily vertical migration of MPB in marine sediments, in the presence of tides or not, it is evident that diel rhythms in primary production in muddy sediments are mainly caused by the changes in autotrophic biomass in the upper sediment layer, at least under the relatively low irradiance used in our experiments. Nonetheless, at high irradiance, physiological regulation of photosynthetic activity would be expected to play a significant role. Additionally, our experiments were done with muddy-silty sediment dominated by epipelic diatoms. Since epipsammic and epipelic diatoms differ in their relative dependence on behavioural or physiological photoprotection mechanisms^33,40^; in sandy marine sediments, where epipsammic diatoms are more abundant, the photophysiological regulation mechanisms (e.g. non-photochemical quenching) might play a larger role in the regulation of diel patterns of PP_MPB_.

The photoperiod is the main contributor to the daily oscillations observed in P_N_ during the light period in intertidal sediments in the absence of any external tidal signal under constant low light irradiance. The P_N_ diel patterns were maintained during several days under continuous light and quickly recovered when the photoperiod was re-established, being convincing proof of the existence of a circadian rhythm. Diel oscillations in P_N_ were the consequence of the vertical migration which was entrained by photoperiod and presented a clear endogenous component as well. However, in the more complex environmental conditions existing in the intertidal zone, light irradiance, the duration of the light period or tidal cycles (immersion/emersion) can likely act as additional environmental signals, altering the amplitude or period of the vertical migration circadian rhythm. From a biological point of view, it is important to be able to identify the relative contribution of the various signals to understand how MPB primary production rate and the vertical distribution of its autotrophic biomass respond to the interactions between these environmental periodic signals, particularly photoperiod and tides. Given the important ecological and biogeochemical role of MPB in shallow environments, these data stress the importance of considering these oscillations when making diel and annual budgets of P_N_ of intertidal systems.

## Material and Methods

### Sediment sampling and general experimental set-up

Sediment (silty mud) and seawater were collected during low tide, from an intertidal muddy area of the inner Cadiz Bay (Trocadero Island, N 36° 30′41.5764″, W 6° 13′59.574″, SW Spain), transported to laboratory and incubated under a 12 L:12D photoperiod at constant irradiance during the light phase (200 µmol photon m^−2^ s^−1^; Lumina 1080 Blau Aquaristic) and temperature (18 °C). Sediment cores were incubated in an aquarium with recirculating seawater from a larger tank. Seawater (30 L) was replaced every three days to maintain nutrients concentrations at *in situ* levels. The taxonomic composition of the MPB community in the sediments used in the experiments was analysed by optical microscopy. The MPB was dominated by epipelic diatoms of the genera *Gyrosigma sp*., *Amphora sp, Achnantes sp. Navicula sp*. and *Cylindrotheca sp*. which represented >95% cells and some minor amounts of cyanobacteria (short chains of *Oscillatoria* sp.). This community was similar to that found in previous studies on MPB in Cádiz Bay^66^.

### Experiments

Three different types of experiments were done to study the diel cycle of primary production and its causes. In the first group of experiments, we determined the diel pattern of net primary production during the light period under constant irradiance (Experiment I, ExI). In the second group, we determined the role of the alternating light and dark phase of photoperiod in the maintenance of the diel photosynthetic rhythm (Experiment II, ExII). In the third group, we studied the coupling between diel net production and vertical migration (Experiment III, ExIII). Irradiance was kept constant at 200 µmol photon m^−2^ s^−1^ during the light phase in all the experiments and cores were always kept in immersion. Reconstituted cores were used in ExI to avoid the risk of possible microsensor breakage, while intact sediment cores were used in the ExII and ExIII to avoid as much as possible any alteration of the migration due to sediment manipulations, despite the risk of possible microsensor breakage.

#### Experiment I

Sediment was collected from two different sediment depths in June 2015: surface (first centimetre) and deep sediment (down to 20 cm depth). Due to large presence of shells, sediment was homogenised and sieved to avoid breaking the microsensors. Transparent plexiglas cores (i.d. = 5.4 cm, n = 6) were filled with the deep sediment (10 cm) and completed with 2 cm of surface sediment to replicate partially the same vertical structure found in the field. Six cores were distributed in three aquaria under a 12 L:12D photoperiod. Cores were pre-incubated for 5 days to allow MPB to grow uncoupled from tides and consequently from any emersion-immersion rhythm. P_N_ and R_d_ were estimated from oxygen profiles measured at the sediment-water interface with O_2_ microsensors every 30 min, during 1 h in darkness, then 12 h in light and finally 1 h in darkness.

#### Experiment II

To test whether the diel rhythm in P_N_ was caused by an endogenous circadian clock or it depended only on photoperiod, intact sediment cores (i.d. = 5.4 cm, n = 4) collected in January 2017 were distributed in two aquaria, one under a 12 L:12D photoperiod for 10 days and another under continuous and constant light for 7 days, followed by a 12 L:12D photoperiod for another 3 days. In addition, we tested the importance of alternating light and dark phases to maintain the diel photosynthetic rhythm and its potential recovery after its disappearance in continuous light when the photoperiod signal was re-established. P_N_ and R_d_ rates were measured as mentioned previously. ExII was repeated independently twice.

#### Experiment III

To test whether the diel changes in P_N_ were related to MPB vertical migration, intact sediment cores (i.d. = 5.4 cm, n = 4) were collected in November-December 2017. Two cores were incubated under a 12 L:12D photoperiod and another two under continuous constant light for 7 days. P_N_ and R_d_ rates were measured as mentioned previously in parallel with the changes in the absorbed light at sediment surface.

### Variable measurements

Oxygen profiles at the sediment-water interface were measured with oxygen selective microelectrodes (Unisense) with a depth resolution of 100 µm^67^. P_N_ and R_d_ were calculated from O_2_ profiles in light and darkness respectively^68^. Changes in MPB biomass at the sediment surface were estimated from the light absorbed by the MPB (A_MPB_), determined from reflectance spectra (350–1000 nm) using a USB-2000 spectrometer (model USB 2000-VIS, Ocean Optics) connected to 1 mm diameter fiber optic (model ZQP400-10-VIS, Ocean Optics)^38,69^. The A_MPB_ was estimated as follows:

$${A}_{sample}=\frac{R708-R663}{R708}=1-\frac{R663}{R708}$$

$${A}_{MPB}={A}_{sample}-{A}_{sediment}$$

Where A_sample_ and A_sediment_ represent the light absorbed by sample and sediment, respectively. A_sediment_ were measured on a filter soaked in pure sediment and positioned on top of the sediment surface at the end of the measurements; R_708_ represent the reflectance at 708 nm and was used as reference value to normalized to reflectance values and remove possible changes of incident light, as no microalgae from the sediment surface has photosynthetic pigments absorbing at this wavelength; R_663_ represent the reflectance at 663 nm which was chosen as an indicator of chlorophyll a, being close to the wavelength where maximum absorption peak of the pure pigment occurs.

Sediment chlorophyll was extracted with methanol and quantified on a spectrophotometer^70,71^. Methodological details can be found in supplementary material.

### Data analysis

A sine wave equation (Supplementary Material, Eq. 6) was fitted on the temporal evolution of net production rate using the Microsoft Excel 2016 Solver add-in to obtain best fit parameters. In addition, a FSA^72^ was applied to extract the dominant frequencies that characterize the P_N_ time patterns under photoperiod and continuous light, to see whether there was any evidence of different frequencies in the time series, i.e. semidiurnal tidal signal, which it might represent a “memory” from *in situ* tides. The coherence and simultaneity between the temporal oscillation in P_N_ and A_MPB_ were tested by CSA^72^. Spectral analyses were carried out by a code expressly designed in Fortran90. Further details for the analysis of data can be found in the supplementary material.

## Supplementary information

Supplementary Material

## Data Availability

All the data are available in the figshare public repository (10.6084/m9.figshare.7110425.v1).
