# Supplementary material for: Diel patterns of microphytobenthic primary production in intertidal sediments: the role of photoperiod on the vertical migration circadian rhythm
Source: Sci Rep. 2019 Sep 16;9:13376. doi: 10.1038/s41598-019-49971-8 (PMC6746711; doi:10.1038/s41598-019-49971-8)

Supplementary material for:

Diel patterns of microphytobenthic primary production in intertidal sediments: the role of photoperiod on the vertical migration circadian rhythm

Haro, S.[1,3*], Bohórquez, J. [1,3], Lara, M.[1,3], García-Robledo[1,3], E., Carlos J. González[2,3], Crespo, J.M.[1], Papaspyrou, S. [1,3] & Corzo, A.[1,3]

[1] Department of Biology, University of Cádiz, Spain.

[2] Department of Applied Physics, University of Cádiz, Spain.

[3] University Institute of Marine Research (INMAR). University of Cádiz, Spain.

*author for correspondence: Sara Haro, email: sara.haro@uca.es

Supplementary information.

Supplementary material and method: variables measurements & data analysis (statistics, sine equation and spectral analysis of net production time series).

Supplementary results: Table S1, Figures S2-4

MATERIALS AND METHODS

Variables measurements

Net primary production and respiration rates

Oxygen profiles at the sediment-water interface were measured with oxygen selective microelectrodes (Unisense) with a depth resolution of 100 µm [1]. Oxygen microelectrodes were connected to a picoammeter (PA2000, Unisense) and the signal was recorded using an A/D converter. The sensor was moved using a motorized micromanipulator connected to a computer controlled using the software SensorTrace Pro v3.0.2 (Unisense). Output signals (mV) were converted to oxygen concentrations (µM) by a linear calibration (100 % air saturation at the water air interface and 0 % at depth in the sediment) for prevailing temperature and salinity conditions ($R^2 > 0.99$).

Net production rates of the photosynthetic layer (P_N) were calculated as the total O_2 flux out of the photic zone, i.e. the sum of upward (J_{up}) and downward fluxes (J_{down}) calculated using Fick's first law on the oxygen vertical profiles [2].

$$P_N = \mathrm{J_{up}} + /\mathrm{J_{down}}/ \quad \text{(eq. S1)}$$

$$\mathrm{J_{up}} \text{ or } J_{down} = -\mathrm{Ds}\,\frac{\mathrm{dC(z)}}{\mathrm{dz}} \quad \text{(eq. S2)}$$

The upward flux was calculated from the slope of the concentration profile (dC(z)/dz) just below the sediment surface within the photic zone, whereas the downward flux was calculated from the slope of concentration profile at the inflection point of O_2 profiles, at the lower limit of the photic zone [2]. The diffusion coefficient of oxygen in the sediment (D_s) was calculated from the molecular diffusion coefficient (D_o) applying a porosity-correction factor of φ^2 [3]. Sediment porosity ($\varphi \approx 0.8$) was determined as the water content loss of a known volume of sediment from the first 0.5 cm sediment layer. Values of D_o were obtained from standard tables [4] calculated [5].

Dark respiration rate was calculated as the oxygen flux across the sediment water interface from profiles in darkness.

$$R_{dark} = -Ds \frac{dC(z)}{dz} \qquad \text{(eq. S3)}$$

When the production peak was very small, as in some profiles measured between 12 am and 6 a.m. in the treatments with constant light in experiments II and III (ExII and ExIII), J_{up} was considered zero and net production rates of the photosynthetic layer were calculated from J_{down} (equation 1). Finally, net production and respiration rates (mmol O_2 m^{-2} h^{-1}) were normalized by the chlorophyll *a* concentration (ExII and ExIII).

Absorbed light by MPB in the sediment surface

Reflectance spectra (350-1000 nm) were measured using a USB-2000 spectrometer (model USB 2000-VIS, Ocean Optics) connected to 1 mm diameter fiber optic (model ZQP400-10-VIS, Ocean Optics). The fiber was positioned obliquely at a fixed distance (about 3 cm) from the sediment surface to measure in the same area where oxygen microsensors penetrated the sediment. Reflectance spectra were measured every 30 min in the light phase during the 7 days experiment (ExIII). Sediment reference spectra were measured on a filter soaked in pure sediment and positioned on top of the sediment surface at the end of the measurements, thus avoiding any light differences due to slight changes in the position of the fiber or sample. In order to normalize and calculate the relative amount of absorbed light, reflectance values were normalized. Light provided by the fluorescent lamps showed two relative maxima at 663 and 708 nm. The reflectance at 708 nm was used as reference value to remove possible changes of incident light, as no microalgae from the sediment surface has photosynthetic pigments absorbing at this wavelength. Absorbed light at 663 nm was chosen as an indicator of chlorophyll *a*, being close to the wavelength where maximum absorption peak of the pure pigment occurs. The relative absorbed light by the MPB (A_{MPB}) was estimated as follows:

$$A_{sample} = \frac{R_{708} - R_{663}}{R_{708}} = 1 - \frac{R_{663}}{R_{708}} \qquad \text{(eq. S4)}$$

$$A_{MPB} = A_{sample} - A_{sediment} \quad \text{(eq. S5)}$$

This approach is similar to the normalized difference vegetation index (NDVI) [6]. The linear correlation between A_{MPB} and NDVI in the experiment IIII was very high ($r^2 = 0.999$). A_{MPB} was calculated from the absorbance at 708 nm as the reference absorbance with no chlorophyll and not at 750 nm as originally suggested [7] because the fluorescent lamps used in the experiments here presented an emission peak.

Chlorophyll concentration

Sediment chlorophyll was extracted from the first 0.5 cm of sediment in 100 % methanol with $MgCO_3$ for 12 hours at 4 °C in darkness [8]. Samples were centrifuged at 5000 rpm for 10 min (Sorvall Legend X1R; Thermo Scientific). The absorbance was measured on a plate reader (Multiskan GO; Thermo Scientific) and converted to concentrations of chlorophyll a [9].

Data analysis

Statistics

Significant differences among respiration rates in darkness were tested using one-way analysis of variance (ANOVA). Relationships between the depth of oxygen penetration, maximum oxygen concentration peak, amount of light absorbed by MPB and net production rates were tested using the Pearson correlation coefficient. Significance level was set at α=0.05 in all cases. Statistical analyses were carried out using Microsoft Excel 2016.

Sine equation

Temporal evolution of net production rate was fitted by sine wave equation (equation S6) using the Microsoft Excel 2016 Solver add-in to optimise the amplitude (A), period (p), phase shift in the height of P_N (s_P) and the overall average value (b) [10,11].

$$P_N = A\ sin\ (\frac{2\pi}{p}(t - s_t)) + b \quad \text{(eq. S6)}$$

Fourier spectral analysis of net production time series

The time series of net primary production (P_N) measured in laboratory were submitted to Fourier spectral analysis (FSA) [12] to determine the relative contribution to the total signal of harmonics with periods of interest [13,14]. The evolution in time of P_N can be expanded into a Fourier series as:

$$P_N(t) = P_0 + \Sigma_{\text{sup}} + P_{24\text{h}} \cos\left(\omega t - G_{24\text{h}}\right) + P_{12\text{h}} \cos\left(2\omega t - G_{12\text{h}}\right) + P_{8\text{h}} \cos\left(3\omega t - G_{8\text{h}}\right) + ... \quad \text{(eq. S7)}$$

Where, P_0 is the time-averaged value; P and G are, respectively, the amplitudes and phase-constants of the different harmonics with periods specified by the subscript; ω is the frequency corresponding to a period of 24 hours; and t is time. The term Σ_{sup} holds for all the harmonics with periods longer than 24 hours; its number depends on the series length. Their corresponding amplitudes were found to be small when resolvable, so they were not further considered. FSA applied to the P_N data series resolved three dominant frequencies at 8, 12 and 24 hours (figure 5). The diurnal frequency (24 h) was 6 times higher than semidiurnal frequency (12 h) under photoperiod treatment and the frequency at 8 hours was only observed under light/dark cycles.

Mathematical model of P_N time series in photoperiod and in continuous light conditions

To investigate if the origin of the different spectral contributions to the total signal, determined by the FSA was ‘physical’ (i.e., due to a real process) or ‘mathematical’ (i.e., due to geometrical properties or nonlinearities of the main signal or a combination of both), we used simple mathematical models of the natural processes involved, adapted to the different light conditions, i.e. alternating 12 h light : 12 h dark photoperiod and continuous light.

Gross production as function of irradiance, P-I curve, was modelled as a hyperbolic relationship without photo-inhibition according to equation S8 [15].

$$P_N = P_{\max}\left(1 - e^{-I/I_k}\right) - R \qquad \text{(eq. S8)}$$

Where, P_{max} is maximum gross production as the irradiance $I \rightarrow \infty$, I_k is the saturating irradiance, and R is respiration. During the experiments in continuous and constant light, the irradiance is time-constant (figure S4) and equal to I_L:

$$I(t) = I_L \qquad \text{(eq. S9)}$$

In the case of the forced-photoperiod experiments, the time-variation of irradiance corresponds to a square-wave function (figure S4) as:

$$I(t) = \begin{cases} I_L \ ; \ 0 < \omega t < \pi \\ 0 \text{ otherwise} \end{cases} \qquad \text{(eq. S10)}$$

According to equation S8, the time series of P_N measured in laboratory, in the absence of other processes, should follow the same pattern as I; i.e., a ‘flat’ wave under continuous light and a square wave under photoperiod. However, the experimental results showed a convex shape (figures 1, 3, 4) during the light period, suggesting that P_N daily change in the constant irradiance during the light phase is controlled by a different process.

The convex shape of periodic oscillation in P_N at a given constant irradiance during the light phase of photoperiod or in continuous constant light can be modelled trough a number of different equations (e.g., polynomial, Gaussian, etc.). Instead, we used a sinusoidal equation (depicted graphically in figure S1) as a first approach to the observed circadian behaviour. In addition, this equation facilitates the analytical expansion of the resulting function into Fourier series.

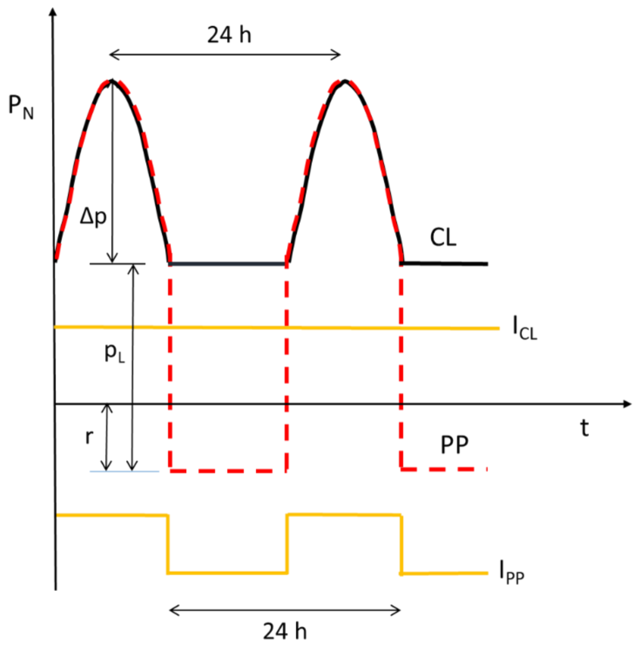

Figure Supplementary 1. Mathematical model for $P_N(t)$ during the laboratory experiments as described by equations S11 and S12, where PP is photoperiod and CL is continuous light.

Hence, the corresponding models for continuous light and photoperiod experiments are, respectively:

Continuous Light: $$P_N(t) = \begin{cases} P_L + \Delta P \sin \omega t - r \,;\, 0 < \omega t < \pi \\ P_L - r \text{ otherwise} \end{cases} \quad \text{(eq. S11)}$$

Photoperiod: $$P_N(t) = \begin{cases} P_L + \Delta P \sin \omega t - r \,;\, 0 < \omega t < \pi \\ -r \text{ otherwise} \end{cases} \quad \text{(eq. S12)}$$

Where, ΔP is the amplitude of the production increase due to vertical migration of MPB respect to the 'basal' production rate P_L corresponding to the laboratory irradiance I_L, and r is respiration. The expansions of equations S11 and S12 into Fourier series give:

Continuous Light:

$$P_N(t) = P_L + \frac{\Delta P}{\pi} + \frac{\Delta P}{2} \sin \omega t - \frac{2\Delta P}{3\pi} \cos 2\omega t - \frac{\Delta P}{15\pi} \cos 4\omega t + \ldots - r \quad \text{(eq. S13)}$$

Photoperiod:

$$P_N(t) = \frac{P_L}{2} + \frac{\Delta P}{\pi} + \left(\frac{2P_L}{\pi} + \frac{\Delta P}{2}\right) \sin \omega t - \frac{2\Delta P}{3\pi} \cos 2\omega t - \frac{2P_L}{3\pi} \sin 3\omega t + \ldots - r \quad \text{(eq. S14)}$$

Equations S13 and S14 allow to evaluate whether the semidiurnal signal (12h) represents a sort of 'tidal memory', since the *in situ* tide is semidiurnal. According to equation S13, the amplitude ratio between the semidiurnal and diurnal contributions in the continuous light experiments is $P_{12h}/P_{24h} = 4/3\pi$ or about 42%, while the observed ratios P_{12h}/P_{24h} obtained from FSA range from 9% to 35%, therefore not higher than the expected mathematical effect. In the photoperiod series, equation S14 gives a theoretical P_{12h}/P_{24h} ratio of $[3(p_L/\Delta p + \pi/4)]^{-1}$. Fitting the experimental data (figure 3) to equation S12 gives a mean $P_L/\Delta P = 0.884$ ($r^2 = 0.953649$), meaning that the contribution of P_{12h} of this 'mathematical' origin is about 30% of P_{24h}. The observed ratios P_{12h}/P_{24h} from FSA range between 6% and 20% in the photoperiod series, being lower than the expected by a mathematical effect. Therefore, the semidiurnal contributions observed in FSA are not higher enough as to represent a true tidal signal in the photoperiod and continuous light data series.

The spectral distributions of the photoperiod P_N time series also show amplitude peaks at periods near 8 hours (third-diurnal) in photoperiod (figure 4). According to equation S14, the expected P_{8h}/P_{24h} ratio is $[3(1 + \pi\Delta P/4P_L)]^{-1}$. When applying the calculated ratio $P_L/\Delta P = 0.884$, the 'mathematical' 8 h contribution should be about 18% of the main diurnal amplitude. Since the observed P_{8h}/P_{24h} ratios provided by FSA ranged from 10% to 25%, there is no clear evidence of an 8 h period contribution different from a mathematical effect. In addition, the absence of clear peaks at periods near 8 hours in continuous light is coherent with equation S13, which does not contain any third-diurnal term at all.

In summary, a clear diurnal contribution is present in all time-series of production both in continuous light and in photoperiod. The semidiurnal and third-diurnal peaks present in the spectral distributions of the measured P_N time series can be explained by the mathematical properties of the production curves, and their effects on the coefficients computed from the Fourier analysis. In the experimental data presented here, we do not find any clear evidence of any potential 'tidal memory' from *in situ* tides reported in other studies.

Supplementary references

1. García De Lomas, J., Corzo, A., García, C. M. & van Bergeijk, S. A. Microbenthos in a hypersaline tidal lagoon: Factors affecting microhabitat, community structure and mass exchange at the sediment-water interface. *Aquat. Microb. Ecol.* **38,** 53–69 (2005).
2. Kühl, M., Glud, R., Ploug, H. & Ramsing, N. B. Microenvironmental control of photosynthesis and photosynthesis-coupled respiration in an epilithic cyanobacterial biofilm. *J. Phycol.* **32,** 799–812 (1996).
3. Ullman, W. J. & Aller, R. C. Diffusion coefficients in nearshore marine sediments. *Limnol. Oceanogr.* **27,** 552–556 (1982).
4. Ramsing, N. B. & Gundersen, J. Seawater and Gases. Tabulated physical parameters of interest to people working with microsensors in marine ecosystems. **Version 2.,** 16 (1994).
5. Li, Y.-H. & Gregory, S. Diffusion of ions in sea water and in deep-sea sediments. *Geochim. Cosmochim. Acta* **38,** 703–714 (1974).
6. Rouse, J. W., Hass, R. H., Schell, J. A. & Deering, D. W. Monitoring vegetation systems in the great plains with ERTS. *Third Earth Resour. Technol. Satell. Symp.* **1,** 309–317 (1973).
7. Serôdio, J., Coelho, H., Vieira, S. & Cruz, S. Microphytobenthos vertical migratory photoresponse as characterised by light-response curves of surface biomass. *Estuar. Coast. Shelf Sci.* **68,** 547–556 (2006).
8. Thompson, R. C., Tobin, M. L., Hawkins, S. J. & Norton, T. A. Problems in extraction and spectrophotometric determination of chlorophyll from epilithic microbial biofilms: towards a standard method. *J. Mar. Biol. Assoc. UK* **79,** 551–558 (1999).
9. Ritchie, R. J. Universal chlorophyll equations for estimating chlorophylls a, b, c, and d and total chlorophylls in natural assemblages of photosynthetic organisms using acetone, methanol, or ethanol solvents. *Photosynthetica* **46,** 115–126 (2008).

10. Mansfield, T. A. & Sanith, P. J. Circadian rhythms. in *Advanced Plant Physiology* (ed. Wilkins, M. B.) 201–216 (Pitman Publishing Limited, 1984).

11. Xu, Y., Ibrahim, I. M. & Harvey, P. J. The influence of photoperiod and light intensity on the growth and photosynthesis of Dunaliella salina (chlorophyta) CCAP 19/30. *Plant Physiol. Biochem.* **106,** 305–315 (2016).

12. Emery, W. J. & Thompson, R. E. Data Analysis Methods in Physical Oceanography. in *Data Analysis Methods in Physical Oceanography.* (eds. Plueddemann, A., Institution, W. H. O. & Woods Hole, M. U.) **15,** 658 (Elsevier Science, 2001).

13. Sorek, M., Yacobi, Y. Z., Roopin, M., Berman-Frank, I. & Levy, O. Photosynthetic circadian rhythmicity patterns of Symbiodium, the coral endosymbiotic algae. *Proc. R. Soc. B Biol. Sci.* **280,** 20122942 (2013).

14. Fukuda, H., Murase, H. & Tokuda, I. T. Controlling Circadian Rhythms by Dark-Pulse Perturbations in Arabidopsis thaliana. *Sci. Rep.* **3,** 1533 (2013).

15. Webb, W. L., Newton, M. & Starr, D. Carbon dioxide exchange of Alnus rubra. *Oecologia* **17,** 281–291 (1974).

SUPPLEMENTARY TABLES

Supplementary Table S1. Average amplitude (A), period (p), phase shift in the height of P_N (s_P) and the overall average P_N value (b) calculated from the fitting a sine wave equation (equation 6) to P_N patterns obtained from sediment cores incubated under a 12 h light : 12 h dark photoperiod or under continuous light in several experiments (data from Ex II (figure 3) and Ex III (figure 4)). Data are mean +/- SE (n=4).

	**12 h L : 12 h D**	**Continuous Light**
**A**	3.05 ±1.38	0.97 ± 0.41
**p**	24.00 ± 0.00	22.92 ± 3.79
**s_t**	7.64 ± 0.20	9.17 ± 0.81
**b**	0.82 ± 0.29	1.28 ± 0.40
**R^2**	0.86 ± 0.02	0.91 ± 0.05

SUPPLEMENTARY FIGURES

Supplementary figure S2. Characteristic changes in the oxygen vertical profiles in submerged intertidal sediment over a diel photoperiod in darkness and under constant irradiance at different times of the light phase (darkness, 1/2 h, 5 h and 12 h after the light is turned on). Max O_2 is the maximum oxygen concentration within the sediment at any given time and z_{ox} is the maximum penetration depth of oxygen.

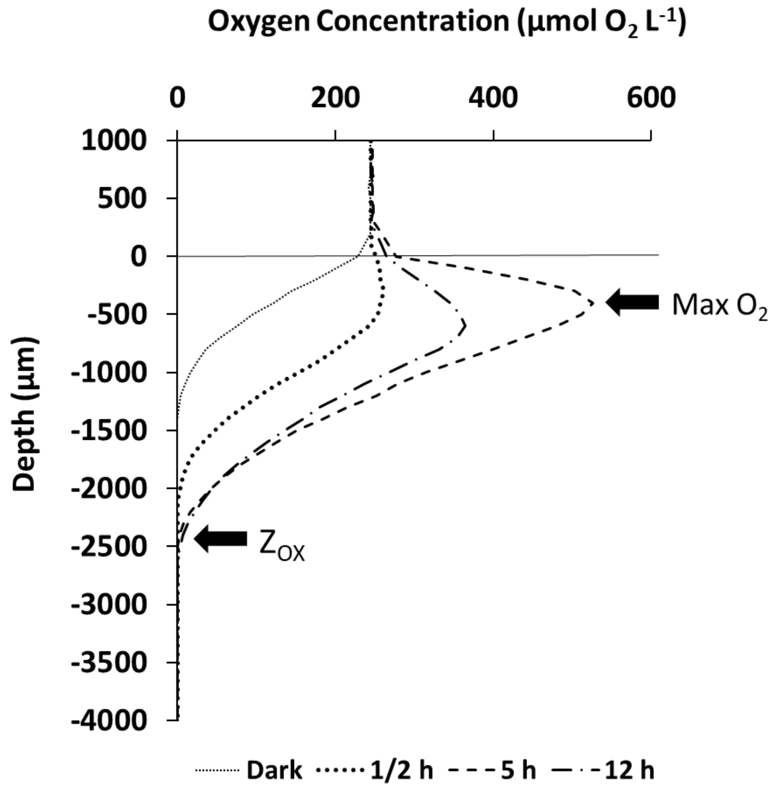

Supplementary figure S3. Decrease of daily maximum net production with time for a 12 h light: 12 h darkness photoperiod (filled symbols) and for continuous light treatment (open symbols). Daily maximum net production was calculated as the average net production between 12.00-16.00 for 4 cores under photoperiod or under continuous light during 7 days. Best fit curves are shown. Values are means ± standard error (SE).

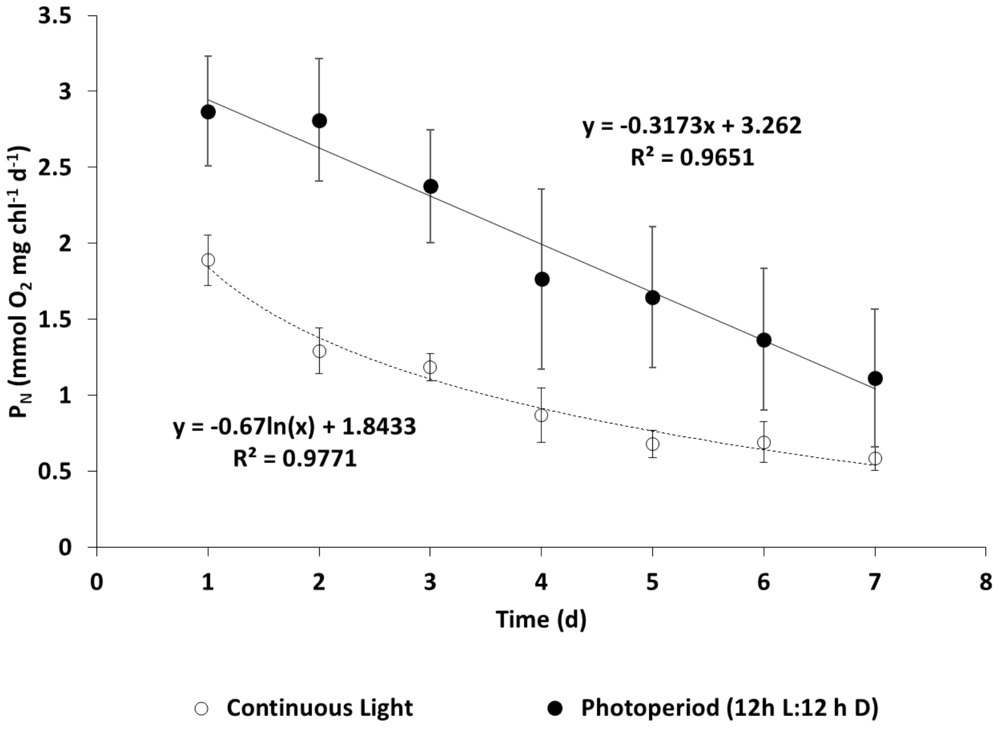

Supplementary figure S4. Relationship between daily maximum P_N and the time difference (in absolute values) between the time of the maximum P_N in the laboratory and the time of maximum low tide in situ. Data belong to experiment II under 12 h L: 12h D photoperiod.

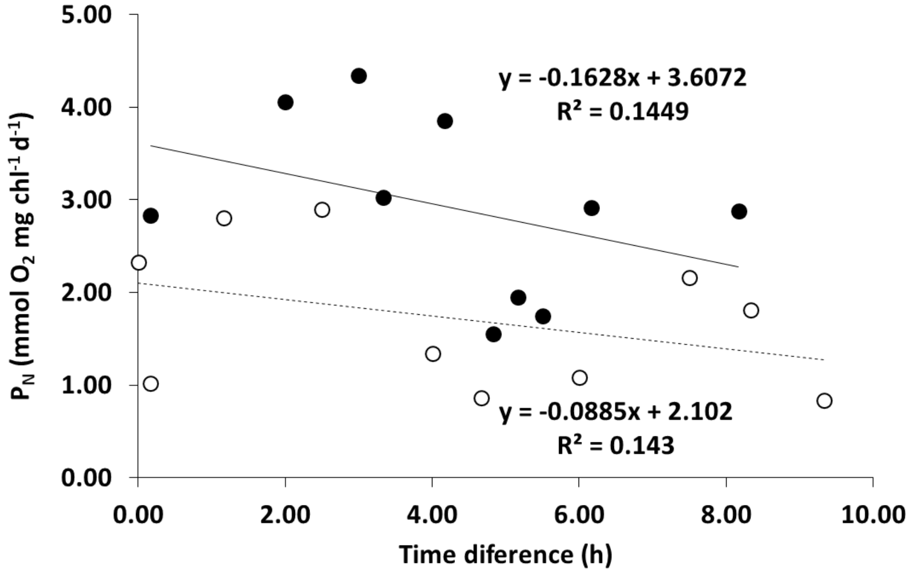

Supplement: Supplementary file 1 — Supplementary Material [file 41598_2019_49971_MOESM1_ESM.pdf]
